# Cardiac-specific methylation patterns of circulating DNA for identification of cardiomyocyte death

**DOI:** 10.1186/s12872-020-01587-x

**Published:** 2020-06-29

**Authors:** Qin Liu, Jian Ma, Hua Deng, Shu-Jun Huang, Jiao Rao, Wei-Bin Xu, Jing-Si Huang, Shan-Quan Sun, Liang Zhang

**Affiliations:** 1 Cardiac center, Guangdong Women and Children Hospital, Guangzhou, 511400 China; 2 Translational medicine center, Guangdong Women and Children Hospital, Guangzhou, 511400 China

**Keywords:** Cardiomyocyte death, Circulating cell-free DNA, Methylation biomarker, FAM101A, qMS-PCR

## Abstract

**Background:**

Correct detection of human cardiomyocyte death is essential for definitive diagnosis and appropriate management of cardiovascular diseases. Although current strategies have proven utility in clinical cardiology, they have some limitations. Our aim was to develop a new approach to monitor myocardial death using methylation patterns of circulating cell-free DNA (cf-DNA).

**Methods:**

We first examined the methylation status of FAM101A in heart tissue and blood of individual donors using quantitative methylation-sensitive PCR (qMS-PCR). The concentrations and kinetics of cardiac cf-DNA in plasma from five congenital heart disease (CHD) children before and after they underwent cardiac surgery at serial time points were then investigated.

**Results:**

We identified demethylated FAM101A specifically present in heart tissue. Importantly, our time course experiments demonstrated that the plasma cardiac cf-DNA level increased quickly during the early post-cardiac surgery phase, peaking at 4–6 h, decreased progressively (24 h) and returned to baseline (72 h). Moreover, cardiac cf-DNA concentrations pre- and post-operation were closely correlated with plasma troponin levels.

**Conclusions:**

We proposed a novel strategy for the correct detection of cardiomyocyte death, based on analysis of plasma cf-DNA carrying the cardiac-specific methylation signature. Our pilot study may lead to new tests for human cardiac pathologies.

## Background

Cardiovascular diseases are the leading causes of death worldwide [1]. Accurate detection of human cardiomyocyte death is crucial for implementing early diagnosis, effective intervention of the disorders. Currently, cardiac-specific troponins (cTns) are widely accepted markers of acute myocardial infarction in clinical practice. However, these biomarkers also have some limitations. Release of troponins into the blood may not necessarily reflect the cardiomyocyte death because cardiac troponins could be released by ischemia alone [2]. For example, healthy individuals after intensive physical exercises, or critically ill patients in intensive care unit may release troponins into circulation leading to elevated troponin levels [3–5]. To date, we do not know whether elevated troponin levels represent cardiomyocyte death, or just reversible cardiomyocyte injury [6, 7]. Moreover, kidney dysfunction can markedly affect troponin clearance [8], which may complicate the explanation of elevated troponin levels in subjects suffered from renal failure. In fact, one of the most important comorbidities in heart failure patients is renal insufficiency [9, 10]. Therefore, development of a new and reliable method is needed to monitor and quantify myocardial death in clinical settings.

Circulating cell-free DNA (cf-DNA), nucleosome-size fragments of genomic DNA in blood, originates from dying cells. In recent years, increased levels of cf-DNA have been found in pathological conditions and clinical diseases such as embryonic chromosomal abnormality [11], tumor prognosis or cancer metastasis [12, 13], inflammation [14], and transplant rejection [15]. Plasma cf-DNA in general may be elevated during tissue injury including cardiomyocyte death and may also be a prognostic indicator of cardiac health [16–20]. However, this phenomenon is not cardiac specific.

Each cell type has a unique DNA methylation pattern, and some tissue-specific DNA methylation biomarkers have been identified [21]. With the advances in methylation analysis, combination of cf-DNA and methylation patterns can trace the tissue origin in circulation [22–24]. More recently, Zemmour et al. found demethylated FAM101A in heart tissues, in contrast to methylated FAM101A in other human tissues including blood. Thus, the unmethylated FAM101A present in a given pool of circulating cf-DNA may be a specific indicator for cardiomyocyte death [25]. Further, they identified high levels of cardiomyocyte cf-DNA in plasma from patients with acute myocardial infarction using droplet digital PCR (ddPCR). In the present study, we used qMS-PCR to determine FAM101A cf-DNA in plasma from five children with congenital heart disease (CHD) undergoing cardiac surgery and concluded that measurement of cardiac cf-DNA levels can indeed detect cardiomyocyte death.

## Methods

### Patients and specimens

The study protocol was approved by the Institutional Review Board of Guangdong Women and Children Hospital and conducted in accordance with the Declaration of Helsinki. Written informed consent was obtained from the individual donors and guardians of all children. We recruited five infants (4 boys and 1 girl, aged 4 days to 2 years) diagnosed with CHD including complete transposition of the great arteries, coarctation of the aorta, coarctation of the aorta with ventricular septal defect into our study. Clinical characteristics for each patient are summarized in Table 1. The main clinical manifestations were cyanosis and dyspnea. After cardiac surgery, all the young patients recovered well and were discharged without complications.

**Table 1:** Clinical information for each patient

Case	Gender	Age	Weight (kg)	Length (cm)	Diagnosis	Clinical symptom	Types of cardiac surgery
1	M	11d	3.8	55	CoA with aortic arch hypoplasia, PFO, PH	Dyspnea	Coarctation repair
2	M	2 m	4	56	CoA with aortic arch hypoplasia, PFO, PH, VSD	Dyspnea, cyanosis	Aortic arch repair, VSD repair
3	M	2y	12.2	81	CoA with aortic arch hypoplasia, VSD (muscular)	Dyspnea after exercise	Aortic arch repair, VSD repair
4	M	4d	3.3	50	TGA/IVS, PDA, PFO	Dyspnea, cyanosis, arrhythmia	ASO, PDA ligation
5	F	13d	3.7	47	TGA/IVS, ASD, PDA, PFO, Ti, PH	Dyspnea, cyanosis	ASO, PDA ligation, ASD repair, unplanned sternum left open

*ASD* Atrial septal defect, *ASO* Arterial switch operation, *CoA* Coarctation of the aorta, *PDA* Patent ductus arteriosus, *PFO* Patent foramen ovale, *PH* Pulmonary hypertension, *TGA/IVS* Transposition of the great arteries with intact ventricular septum, *Ti* Tricuspid insufficiency, *VSD* ventricular septal defect

Since atrial appendage is a remnant of the primitive atrium and it does not affect the heart functions, atrial appendage tissue (5 × 5 mm) was taken from a patient with atrial fibrillation during surgery to assess the specificity of the cardiac methylation biomarker. Peripheral blood samples were collected from CHD patients before and after they underwent cardiac surgery at serial time points in Cell-Free DNA Storage Tube containing EDTA (Genphar Company, Guangzhou, China) for methylation analysis of FAM101A cf-DNA. In addition, the same samples were subjected to measurement of serum levels of cTn-I.

### DNA extraction

Genomic DNA from heart tissue was isolated using the TIANamp Genomic DNA Extraction Kit according to the manufacturer’s protocol (Tiangen Biotech, Beijing). Whole blood was centrifuged quickly at 1350 ± 150 RCF for 10 min at room temperature. Plasma was carefully transferred to a new tube and then stored at − 20 °C until DNA extraction. Cell-free DNA was isolated from plasma using the Nucleic Acid Purification/Magnetic Beads Kit (GENESHINE, Shanghai, China) following the manufacturer’s recommendations. DNA concentrations were quantitated using the Qubit® dsDNA HS Assay Kit (Thermo Fisher Scientific).

### Quantitative methylation-sensitive PCR (qMS-PCR)

**Fig. 1:**
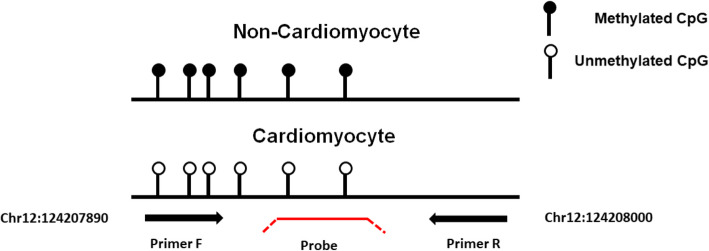
Schematic diagram of the primer set and probe designed for qMS-PCR. Lollipops represent CpG sites of FAM101A locus and arrows mark positions of primer set

The isolated cf-DNA was treated with bisulfite using the GS DNA Methylation Kit (GENESHINE, Shanghai, China). The bisulfite-converted DNA was immediately subjected to a qMS-PCR assay. The cycling conditions were 94 °C for 10 min, followed by 50 cycles of 93 °C for 30 s, 56 °C for 1 min and 65 °C for 30 s, ended by 40 °C for 30 s. All analyses were performed on an ABI 7500 Fast Real-Time PCR system (Applied Biosystems) and tests for each sample were performed in triplicates. The house keeping gene beta actin (ACTB) was measured as an internal control. The cycle threshold **(**Ct) value for ACTB less than 32 ensured good sample quality and indicated the validity of the testing**.** We measured the Ct values for FAM101A and ACTB in each sample and the DNA methylation level was calculated based on the differences between the two Ct values (ΔCt = Ct ^FAM101A^*–* Ct ^ACTB^). To optimize the performance of the assay, a △Ct value < 9 for positive testing was established in our pilot experiments. We designed forward primer to cover 3 CpG sites (CpG 1–3) in the cluster, probe for CpG 5, 6 and reverse primer located downstream of the locus using Primer premier 5.0 (Fig. 1). The length of the amplified product was 144 bp. The following primer and probe sequences were used for qMS-PCR:

FAM101A-F:5′- ATGAT*TG*ATAATAA*TG*TA*TG*GTG − 3′ (CpGs in italics).

FAM101A-R:5′- CCTCCACAAAATTTACCT -3′.

FAM101A-P:5’FAM- T*CA*ACTTCCATCTA*CA*ATTCCCA -3′ MGB (CpGs in italics).

ACTB-F: 5′-TGGTGATGGAGGAGGTTTAGTAAGT-3′.

ACTB-R: 5′-ACCAATAAAACCTACTCCTCCCTTAA-3′.

ACTB-P: 5’HEX- ACCACCACCCAACACACAATAACAAA CACA-3’BHQ1.

### Measurement of cTn-I

Quantitative measurement of cTn-I in serum was conducted using a commercially available fluorescence immunoassay (M101–091011^#^, MicroPointBio, Shenzhen, China) according to the manufacturer’s recommendations. The concentration of each analyte in the specimen is directly proportional to the fluorescence intensity.

## Results

### Identification of unmethylated FAM101A in heart tissue

**Fig. 2:**
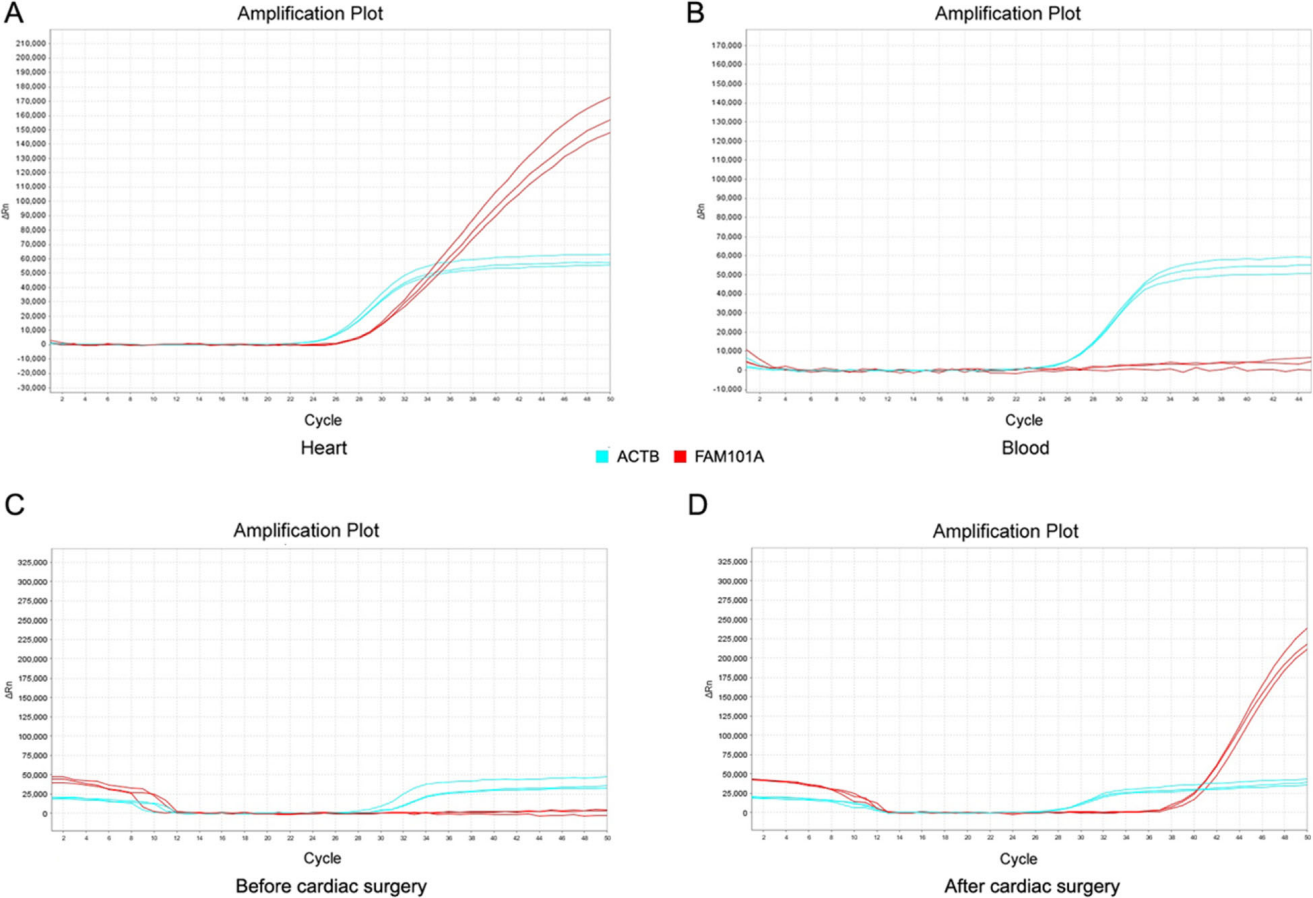
Quantitative methylation-sensitive PCR amplification curves for FAM101A. Heart tissue (**a**) and blood (**b**) from individual donors; Plasma cf-DNA pre-operation (**c**) and post-operation (**d**) from patients with congenital heart diseases

To assess the specificity and utility of the cardiac methylation biomarker in our qMS-PCR assays, we analyzed the methylation status of FAM101A in known positive (heart tissue with demethylated FAM101A) and negative (blood with methylated FAM101A) samples based on a prior study. As expected, strong amplification signal was detected in heart tissue, whereas a negative result was found in blood (Fig. 2a-b), indicating the validity of the new approach.

### Methylated FAM101A DNA in the circulation confirmed by sanger sequencing

**Fig. 3:**
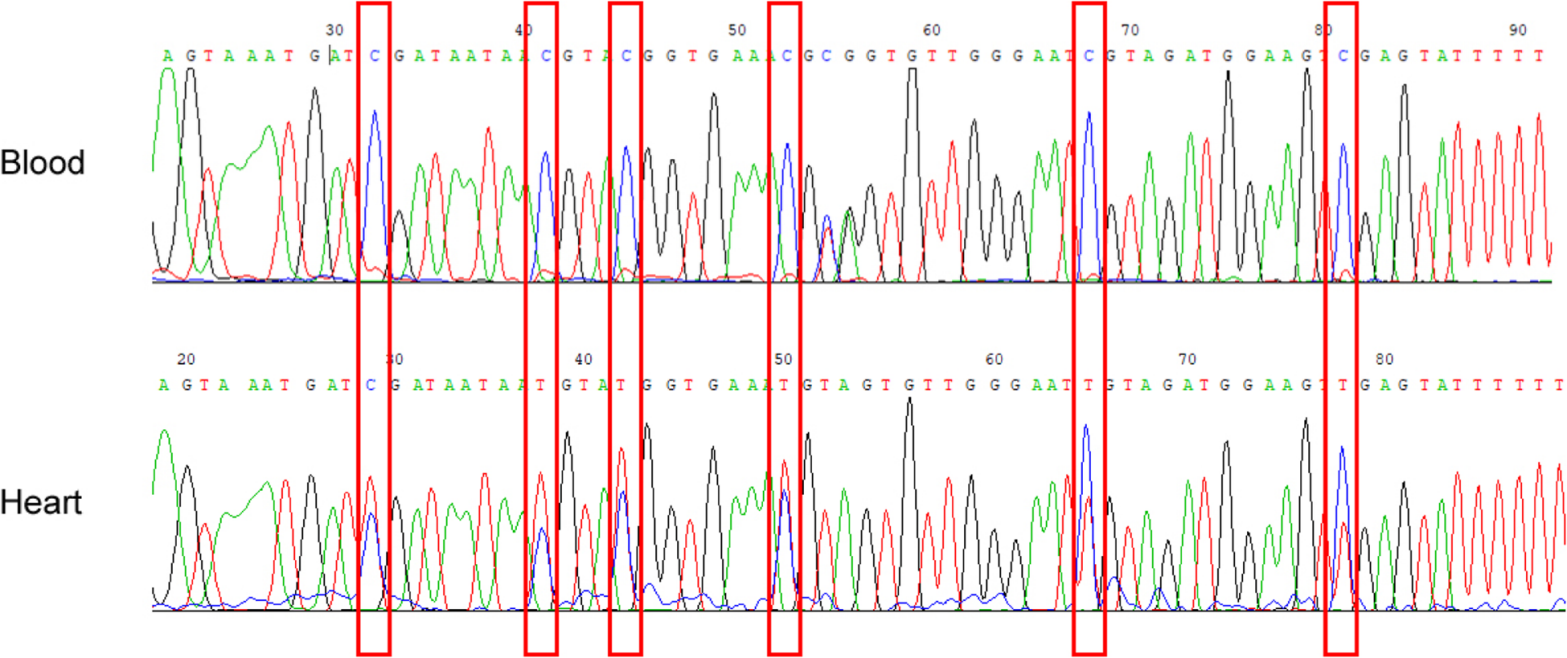
Sanger sequencing of the PCR products. Sequencing chromatograms for genotyping of FAM101A promoter exhibited a homozygous (single) peak at each targeted CpG site in blood, and heterozygous (double) peaks in heart tissue

To ascertain the methylation status of FAM101A in heart tissue and blood, we designed another pair of primers (FAM101A-F: ATGGATAAGGAAATTAAGATAG, FAM101A-R: AAATACAAATCCCACAAATAAA) to recognize the regions outside the CpG sites of FAM101A promoter in a regular PCR assay. The resulting PCR products generated from both blood and heart tissue were subjected to Sanger sequencing. Sequencing chromatograms exhibited homozygous base (C) at each targeted CpG site in blood (Fig. 3). However, heterozygous bases (T/C) at all six CpG sites were present in heart tissue. This could be ascribed to the fact that heart tissue contains non-cardiac tissues/cells such as vascular and connective tissues. The difference in DNA sequence of FAM101A promoter between the two samples was reliant on the methylation status at the targeted CpG sites.

### Identification of cardiomyocyte death in cf-DNA from patients after cardiac surgery

**Fig. 4:**
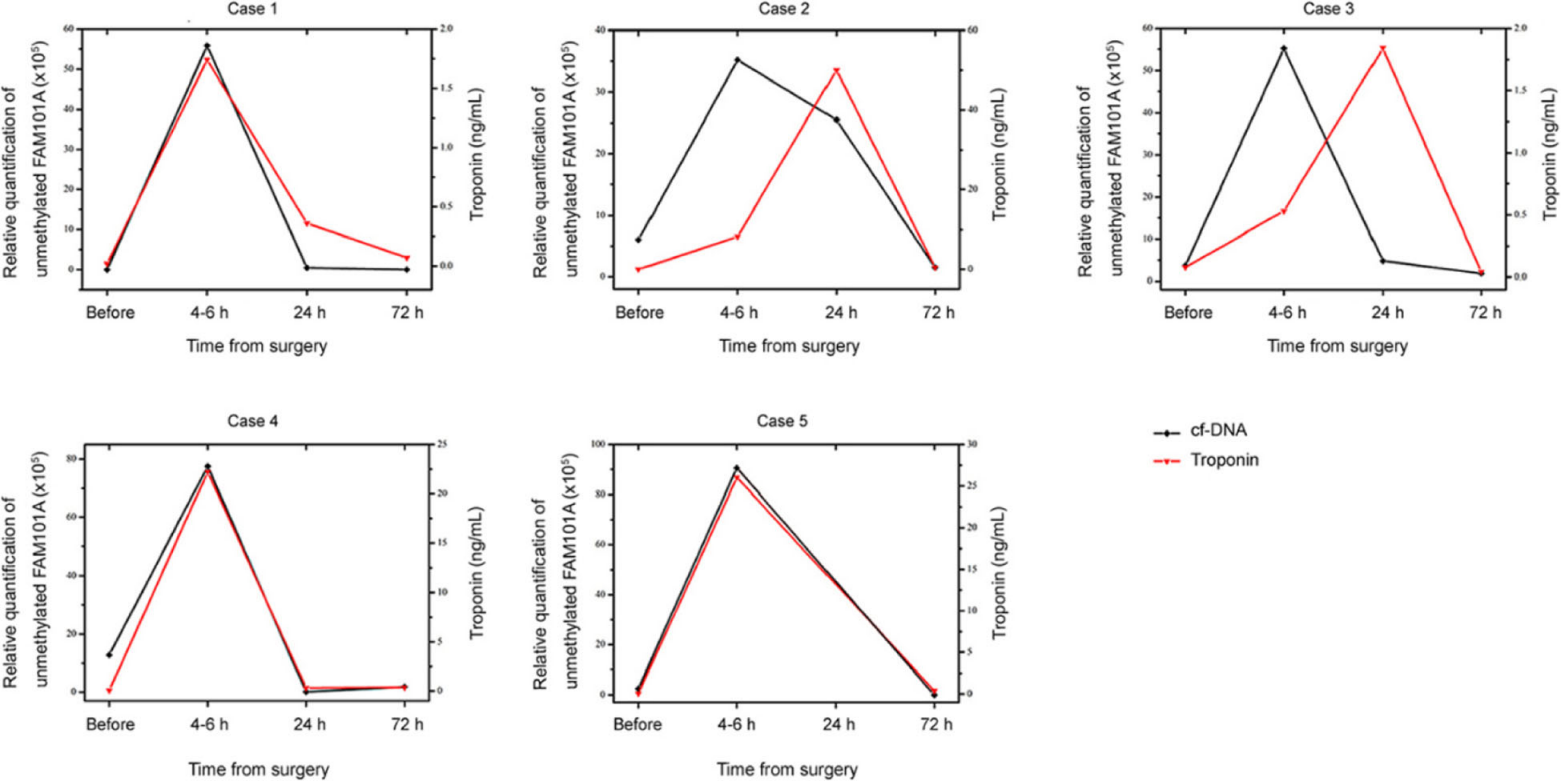
Temporal course of cardiac cf-DNA and troponin in five patients undergoing cardiac surgery

Further, we tested whether the new method could be useful in identifying cardiomyocyte death in circulating cf-DNA. To this end, we investigated the plasma levels of cf-DNA in CHD children before and after cardiac operation. Samples with a △Ct value (Ct^FAM101A^-Ct^ACTB^) < 9 were considered to represent a positive test result. Overall, similar findings were observed among the five subjects and determining the cardiac cf-DNA levels can indeed detect cardiomyocyte damage (Fig. 2c-d). To further examine the temporal course of cardiac cf-DNA, we analyzed the concentrations of plasma demethylated FAM101A before and after cardiac surgery at serial time points. As shown in Fig. 4, we identified the low levels of cardiac cf-DNA pre-operation, a large increase at 4–6 h post-cardiac surgery, followed by a progressive decrease to baseline (24 h - 72 h).

### Correlation of plasma cf-DNA with cardiac troponin levels

To compare the new molecular signature with classic cardiac biomarker, cTn-I was also examined with a fluorescence immunoassay. Both cardiac cf-DNA and troponin levels were elevated in all instances of post-operation, and then decayed to a low level. Overall, cTn-I levels correlated well with cardiac cf-DNA concentrations at serial time points (Table 2 and Fig. 4). The findings demonstrated the suitability and robustness of the novel biomarker for non-invasive monitoring cardiomyocyte death.

**Table 2:** Comparison of plasma cardiac cf-DNA and troponin levels in patients before/after surgery

Case	Gender	Diagnosis	Time points	Demethylated FAM101A	cTn-I
1	Male	CHD	Before cardiac surgery	–	–
			4–6 h after cardiac surgery	+	+
			24 h after cardiac surgery	–	–
			72 h after cardiac surgery	–	–
2	Male	CHD	Before cardiac surgery	–	–
			4–6 h after cardiac surgery	+	+
			24 h after cardiac surgery	+	+
			72 h after cardiac surgery	–	–
3	Male	CHD	Before cardiac surgery	–	–
			4–6 h after cardiac surgery	+	+
			24 h after cardiac surgery	–	+
			72 h after cardiac surgery	–	–
4	Male	CHD	Before cardiac surgery	–	–
			4–6 h after cardiac surgery	+	+
			24 h after cardiac surgery	–	–
			72 h after cardiac surgery	–	–
5	Female	CHD	Before cardiac surgery	–	–
			4–6 h after cardiac surgery	+	+
			72 h after cardiac surgery	–	–

*CHD* Congenital heart disease, *cTn-I* Cardiac troponin-I

## Discussion

Plasma cf-DNA is derived from dying cells and many human cells can release DNA into the blood. Thus, circulating cf-DNA is a composite of multiple heterogeneous cell origins [26]. DNA methylation is an ideal indicator for source tissue of plasma cf-DNA because it reflects cell identity. A prior work revealed that demethylated FAM101A was found only in heart tissues using ddPCR [25]. Since ddPCR is quite expensive and complicated, whereas qPCR is a routine technique in molecular labs, we thus established a qMS-PCR assay to trace cardiomyocyte death during the perioperative period via methylation analysis of FAM101A in plasma cf-DNA. Using ACTB gene as an internal reference, we can perform relative quantitation of FAM101A methylation in a simple and easy way. Elevated cardiac cf-DNA was determined in blood from patients after cardiac surgery, indicating the ability of the new strategy that allows inference of cellular origins of circulating cf-DNA in a specific manner. Our study may expand the clinical applications of the cardiac-specific methylation patterns of circulating cf-DNA. The new biomarker should find utility in diagnosis and monitoring of cardiac pathologies.

In a previous study, Zemmour et al. have comprehensively checked the methylation status of FAM101A in 23 other human cell types/tissues including hepatocytes, B cells, breast luminal cells, breast myoepithelial cells, fibroblasts, neutrophils, monocytes, keratinocytes, adipose tissue, muscle, aorta, adrenal gland, pancreas, liver, lung, small intestine, spleen, esophagus, colon, thymus, ovary, hippocampus and stomach [23]. They found that heart tissues (right atrium and left/right ventricle) had dramatically higher levels of demethylated FAM101A compared with 23 other human tissues, indicating an excellent cardiac specificity of FAM101A methylation in the tissue panel examined. Since they have already done that work, we used heart tissue as a positive sample with high levels of cardiac specific DNA and blood as a negative control without cardiac cf-DNA to confirm the validity and specificity of the new approach.

Because heart tissue contains non-cardiac components such as blood vessels and connective tissues, even blood cells, during a biopsy procedure, thus the heterozygous bases (T/C) at each targeted CpG site were observed in heart by Sanger sequencing. From Fig. 3, only methylated FAM1010A molecules corresponding to the homozygous base (C) at CpG sites was present in blood from a healthy donor. That is critically important in this testing system.

It is not strange for the great individual differences about the cTn-I and cardiac cf-DNA levels among five subjects. Compared with myocardial infarction and ischemic cardiomyopathy, cardiac surgery should be a bigger strike or stress for heart tissue and likely causes the levels of cTn-I and cardiac cf-DNA to soar. In addition, different types of cardiac surgery for various congenital heart diseases may result in distinct heart damage. In fact, great individual differences in cTn-I (2–10 ng/mL) and cardiac cf-DNA (120–10,000 copies/mL) levels were also found in patients with acute ST-elevation myocardial infarction after primary percutaneous coronary intervention in a recent study [23].

Moreover, we also examined the time course of plasma cf-DNA pre- and post-operation. We identified a large increase of cardiac cf-DNA at 4–6 h after cardiac surgery and decreased progressively (24 and 72 h) in all five patients. More importantly, we found that the overall kinetics of cardiac cf-DNA were closely correlated with that of cTn-1. For case 2 and case 3, there is a clear shift in the curves for plasma cf-DNA vs. troponin, whereas in all the others the curves are well aligned. The discordant curves may reflect differences in the release and/or clearance rates of cf-DNA and troponin. The observations appear to support the concept that the half-life of circulating cf-DNA is thought to be short, and they are quickly removed by the liver [27]. Plasma cf-DNA may possess a faster response to cardiomyocyte death compared with troponin. Take case 3 for example, it showed high level of demethylated FAM101A and still low level of troponin at 4–6 h after cardiac surgery. Elevation in cf-DNA preceded increase in troponin levels, which actually underscores the clinical value of cf-DNA for monitoring the onset and progression of cardiomyocyte death, potentially complementing currently available markers.

This study has some limitations. First, the main issue of the new method is the turnaround time due to the time-sensitivity of diagnostics in cardiology. Our sample-to-answer turnaround time was approximately 8 h. Further experiments are required to optimize the procedures. Second, the sample size is small. However, despite the small number of cases studied, plasma cardiac cf-DNA analyses could accurately discriminate the presence or absence of cardiomyocyte death. Other limitations include unequal gender balance (only one female patient, four male cases), and the young ages of the individuals (< 2 years) so that we cannot assess the performance of cardiac cf-DNA in older subjects. In addition, a potential shortcoming is the rather narrow evaluation under cardiac surgery as opposed to other conditions, which are more responsible for myocardial cell death in the population, e.g. myocardial infarction. Further testing of the cardiac-specific methylation marker in clinical settings is warranted.

## Conclusions

In summary, we developed a novel method to accurately detect cardiomyocyte death in plasma cf-DNA by analyzing the methylation status of FAM101A with qMS-PCR. Our study adds new insights into this field, and may have a wider application in myocardial pathologies.

## Data Availability

The data set used and/or analyzed during the current study is available from the corresponding author on reasonable request.

## Abbreviations

cf-DNA: Cell-free DNA
cTns: Cardiac-specific troponins
CHD: Congenital heart disease
qMS-PCR: Quantitative methylation-sensitive PCR
